# Unattended Home Labor until Complete Cervical Dilatation Ending with Hospital Delivery: Analysis of 238 Pregnancies

**DOI:** 10.1155/2013/196709

**Published:** 2013-02-26

**Authors:** Ozlem Gun Eryilmaz, Nasuh Utku Dogan, Cavidan Gulerman, Leyla Mollamahmutoglu, Nedim Cicek, Ruya Deveer

**Affiliations:** Zekai Tahir Burak Women's Education and Research Hospital, 62000 Ankara, Turkey

## Abstract

*Objectives*. Hospital fear and avoidance of the routine hospital obstetrical interventions cause some women with low-risk pregnancies to spend most of the active labor period at home, and subsequently they present to the hospital for delivery. Our aim was to analyze the maternal and neonatal outcomes of pregnancies with a planned hospital birth, yet spending the first stage of labor at home without a health provider and completing the delivery in the hospital setting. *Methods*. We retrospectively compared 238 pregnancies having home labor plus hospital delivery (study group) with 476 pregnancies that had spent the whole labor in the hospital setting, considering various maternal and neonatal outcomes. *Results*. Cesarean and episiotomy rates were lower (*P* < 0.0001 and *P* < 0.001, resp.), but neonatal intensive care unit admissions of the infants were more prevalent (*P* < 0.01) in the study group. Other maternal and neonatal outcomes including neonatal mortality were comparable. *Conclusion*. Although our preliminary data generally do support the safety of home active labor plus hospital delivery for low-risk pregnancies, the clinical implications of current data warrant further prospective trials.

## 1. Introduction

Hospital setting is generally considered the safest place for labor and delivery due to the presence of qualified birth attendants, specialized personnel, and modern technological equipment. There are several studies focusing on the safety of a planned home birth, in which the maternal and neonatal outcomes are comparable with hospital births [1–11]. Actually, the fact is the decreasing home birth rate around the world despite relatively favorable results following home birth [12].

The woman's decision about the place of labor is affected by many factors. Feelings of being safe, comfortable home environment, and the assistance of the relatives may provide some women with more physiological support in a home than a hospital birth. Additionally, the geographical distance between home and emergency services could be a distressing factor in the presence of possible complications.

Turkish maternity care system generally favors hospital birth, aiming to decrease the maternal and neonatal mortality rates. In urban regions, most of the births take place in hospitals with the attendance of an obstetrician. However in certain rural districts, home births with or without the attendance of healthcare personnel are still the leading category of birth. There is another group of women, who belong to none of the previously-mentioned groups. They plan a hospital birth, but spend most of the labor period at home outside the followup of any healthcare personnel and subsequently complete the delivery in the hospital setting. Latent and active labor periods in the first stage are completed at home, and they would eventually present to the hospital labor ward with complete or near-complete cervical dilatation and effacement. Therefore, the active labor period that is spent at home is considered “alone.” This implies absence of healthcare personnel during labor, indicating that the woman was alone regarding any professional healthcare support or obstetrical intervention. However, the laboring woman's partner, relatives, or neighbors are the only attendants in this scenario. Consequently, the delivery takes place in the hospital setting mostly with an attending obstetrician.

The implications of home labor followed by hospital delivery have not been adequately studied, and data on these pregnancies are scarce. In the current study, our aim was to analyze the maternal and neonatal outcomes of pregnancies with home labor plus delivery in our hospital. 

## 2. Materials and Methods

We conducted a retrospective case control study between January 2008 and September 2009 in Zekai Tahir Burak Women's Education and Research Hospital, Ankara, Turkey. This hospital is a tertiary center specialized in women's health. Using hospital admission data, 279 women were found to present to the labor ward with full dilatation, and of these 238 were singleton and low-risk pregnancies (≥37 weeks' gestation) that comprised the study group. Exclusion criteria were (i) multiple pregnancies, (ii) previous caesarean section, (iii) preterm deliveries (<37 weeks), and (iv) high risk pregnancies including (but not limited to) hypertension, diabetes, other endocrinological problems, and abnormal placental localization.

Women in the study group had spent the first stage at home presented to the emergency room with complete cervical dilatation at the end of the active first stage, and delivery was performed in the hospital. A total of 476 gestational-age matched pregnancies whose active labor period and the delivery were spent in the hospital setting set up the control group. Relevant data were extracted from maternal and neonatal files.

Groups were compared considering maternal age, parity, antenatal follow-up characteristics, fetal presentation, signs of labor, presence of meconium-stained amniotic fluid, route of delivery, and maternal complications including episiotomy requirement. The analyzed neonatal outcomes comprised of birth weight, Apgar scores at minutes 1 and 5, and the requirement for admission to the neonatal intensive care unit (NICU).

Additionally, data on the causes of prolonged home period of the labor were also specifically retrieved if accessible. Preference for this conduct was grouped as (1) desire for a relaxed environment during labor, (2) hospital fear, (3) logistic problems, that is, transportation to the hospital, and (4) woman's preference for her partner's attendance during transfer to the hospital.

Kolmogorov-Smirnov tests with histograms and P-P plots were used to evaluate the normality of the continuous variables within groups. Mann-Whitney *U* test and independent sample *t*-test were used to compare continuous variables. For categorical data, Pearson's chi-square or Fisher's exact test was utilized for comparisons. A *P* value less than 0.05 was considered significant.

## 3. Results

 The incidence of home labor plus hospital delivery was 0.7% within all the deliveries during the study period. Demographic data and the maternal outcomes of the study and control groups are presented in Table 1. The parity was significantly higher in the study group (Table 1). Women with home labor were more likely to have received no antenatal followup compared to controls (Table 1). The labor signs were also different between the two groups. Both groups had abdominal pain as the mostly pronounced complaint. The rate of painful uterine contractions was 95.4% for home labor and 71.4% for the hospital labor groups (*P* < 0.01). Rupture of the membranes was higher in the hospital labor group than that of the home labor group (18.3% versus 0.8%, *P* = 0.0001), and bleeding was more prevalent in the hospital labor group (3.8% versus 11.3%, *P* = 0.0001). 

The mean gestational ages at presentation were similar across the groups. The requirement for episiotomy and cesarean delivery was significantly lower in the study group (Table 1). Other perinatal complications including meconium-stained amniotic fluid were comparable with no significant differences (Table 1). There was a single postpartum uterine atony case (0.4%) in the home labor group versus two women with postpartum hypotonic uterus and one placental bed bleeding (0.4%). 

The neonatal outcomes of the groups are given in Table 2. Mean birth weights and Apgar scores did not differ among the groups. Analyses of neonatal intensive care unit (NICU) requirements for the newborns are summarized in Table 3. Infants born after home labor were more likely to be admitted to NICU (Table 2). Respiratory depression was the most pronounced neonatal problem (44%) in the NICU in the study group. There were no neonatal mortalities in the home labor group versus one in the hospital labor group due to probable birth asphyxia (not significant). 

The motives for spending the first stage of labor out of the hospital environment are summarized in Table 4. Most common explanations were hospital fear caused by the negative birth experiences (46%) and desire to stay longer in the so-called comfortable home conditions (44%).

## 4. Discussion

 Various investigations [1–6, 8, 10] on the feasibility of home birth have reported relative safety and convenience of home labor and delivery if attended by a certified healthcare provider, mostly a midwife. Many countries show a decline in home birth rates except the Netherlands, the leading country with a home birth rate of 30.3% [5]. The Turkey maternity care system encourages planned hospital births. In urban regions of the country, hospital birth attended by an obstetrician is generally the standard. However, the study population in the current series was apparently diverse in that the first stage of labor period was outside the hospital, despite the location of the hospital in an urban residential area.

Our data revealed that home labor was not a decision of a specific age group in the defined population. Both adolescent and middle-aged pregnant women had similar intentions for choosing to spend most of the time for active labor period at home. Close social contacts such as the elder relatives and neighbors may affect the younger inexperienced pregnant women. The older women's orientations possibly direct them to home labor behavior, reflecting their proposals to younger generations. On the other hand, multiparous pregnant women with uncomplicated previous birth experiences could be courageous about the home labor. This subset of women probably do not have “hospital fear,” but prefer the more comfortable home environment, as reported in other studies on home births [13–15]. The more experienced the pregnant woman about birth, the higher the home labor rate was reported. For instance, home birth frequency was found to be two times much more common among multiparous women with a low-risk previous pregnancy [5].

Previous negative birth experiences of the pregnant woman and/or other close social contacts also seem to lead to delayed presentation at the labor ward. These findings were parallel to previous information on home birth [13–16]. A planned hospital birth actually would not indicate an entirely hospital birth experience in the current study; in fact most of the time in labor had been bypassed at home so as to not experience the negative hospital practices. These women probably consider that home was the safest, most comfortable and relaxing environment during the active labor period. It is obvious that further investigations are needed to figure out the social and medical motivations of such behavior in those women.

Women preferring home labor were also more likely to fail to comply with a regular antenatal followup. It is possible that supposedly low-risk pregnancy profile led these women not to attend regular antenatal followup. Alternatively, women vigilant about the antenatal visits also prefer the complete hospital labor and delivery attended by an obstetrician. This might be related to a proposal that medical interventions and routine hospital procedures are necessary and medical interventions worth to have a healthy birth process.

Signs of labor, such as vaginal bleeding, amnion leakage, or abdominal pain, seemed influential for the women in the study to remain at home. Rupture of the membranes was almost absent in the home labor women. On the other hand, vaginal bleeding was the leading sign in the hospital labor group. Probably, vaginal bleeding was considered as an emergency sign to reach the physician. Vaginal bleeding or amnion leakage is apparently a more objective symptom that most women and their partners would regard as an emergency during birth. Women free of these signs can be proposed to feel more stress-free and safe at home, formulating home a more logical environment for them. 

Decreased caesarean section rate in the study group can partially be explained by the lack of labor induction and augmentation. Pregnancies with an unattended first stage of labor would receive less medical interventions. This might account for lower cesarean rates in this subset of women. Meconium-stained amniotic fluid risk was not increased in the home labor group. It is possible that lack of labor induction also prevented unnecessary episiotomies in our population. It can be hypothesized that physiological relaxation capacity of the pelvic floor muscles was more pronounced in women with home labor. 

Interestingly, assistance by healthcare personnel during the active first stage did not negatively affect most maternal and neonatal complications in our data. Despite medically nonsupervised labor, infants of the study group had similar Apgar scores at 1 and 5 minutes compared with controls. However, increased NICU admissions of the home labor group were mostly related to respiratory problems with no significant effect on neonatal mortality. These could indicate that the respiratory problems of infants from those low-risk pregnancies were not associated with increased mortality, probably indicating relative safety of absence of professional labor followup at least in some women. Nevertheless, this information should be evaluated with caution, as we do not have data on medium- to long-term neonatal outcomes. The number of the pregnancies we included might also be undersized to draw straightforward conclusions for relatively uncommon outcomes such as various neonatal problems. 

In conclusion, our preliminary analyses on the maternal and neonatal outcomes of low-risk pregnancies with the out-of-hospital first stage of labor in the absence of any health personnel revealed that such approach might be relatively safe. However, requirement for NICU following delivery is a concern in infants of these pregnancies. 

## Figures and Tables

**Table 1:** Comparisons of  demographic data and pregnancy outcomes of  pregnancies with home and hospital labor.

	Home labor group (*n* = 238)	Hospital labor group (*n* = 476)	*P* value
Age (years)	26 ± 5.4	25.4 ± 5.1	NS
Parity	1.2 ± 1.0	0.7 ± 0.9	0.0001
Prenatal care (%)	55.5 (132/238)	74.4 (254/476)	0.0001
Gestational age (weeks)	38.7 ± 1.2	38.9 ± 1.0	NS
Fetal presentation (head) %	96.2 (228/238)	99.4 (473/476)	NS
Meconium in amniotic fluid (%)	2.9 (6/238)	2.7 (12/476)	NS
Episiotomy rate (%)	50.4 (119/238)	62.2 (296/476)	0.001
Cesarean delivery rate (%)	5 (11/238)	17.1 (81/476)	0.0001
Composite morbidity (%)	4 (9/238)	4 (19/476)	NS

Values are expressed as mean ± standard deviations. NS: not significant.

**Table 2:** Comparisons of  certain neonatal outcomes of  pregnancies with home and hospital labor.

	Home labor group (*n* = 238)	Hospital labor group (*n* = 476)	*P*
Birth weight (g)	3284.9 ± 427.9	3303.1 ± 432.3	NS
Apgar score at minute 1	6.9 ± 0.3	6.9 ± 0.1	NS
Apgar score at minute 5	8.9 ± 0.3	8.9 ± 0.0	NS
NICU admission (%)	7.6 (18/238)	2.5 (11/476)	0.01

Values are expressed as mean ± standard deviations. NICU: neonatal intensive care unit. NS: not significant.

**Table 3:** Details of  outcomes of  the infants admitted to the neonatal intensive care unit in the study and control groups.

	Home labor (*n* = 18)	Hospital labor (*n* = 17)
Respiratory morbidity (%)	44.4 (8/18)	58.8 (10/17)
Meconium aspiration syndrome (%)	16.6 (3/18)	5.8 (1/17)
Major congenital abnormality (%)	5.5 (1/18)	0 (0/17)
Anemia (%)	0 (0/18)	5.8 (1/17)
Polycythemia (%)	16.6 (3/18)	0 (0/17)
Hyperbilirubinemia (%)	11.1 (2/18)	29.4 (5/17)
Caput succedaneum (%)	5.5 (1/18)	0 (0/17)
Neonatal mortality (%)	0 (0/18)	5.8 (1/17)

For all comparisons, *P* > 0.05.

**Table 4:** Grounds for late hospital admissions in the study group.

	Home labor group (*n* = 238)
Hospital fear (%)	46.2 (110/238)
Remain at home during labor (%)	44.5 (106/238)
Woman waiting for her partner (%)	6.3 (15/238)
Logistic problems: home far from hospital (%)	2.9 (7/238)
